# Physiological and Molecular Responses of *Zoysia japonica* to Rust Infection

**DOI:** 10.3390/ijms23084185

**Published:** 2022-04-10

**Authors:** Di Zhang, Jun Tang, Kai Wei, Shangang Jia, Yiwei Jiang, Hongwei Cai, Peisheng Mao, Manli Li

**Affiliations:** 1 College of Grassland Science and Technology, China Agricultural University, Beijing 100193, China; ldichuengcau@163.com (D.Z.); tangjun1@genomics.cn (J.T.); weik17@lzu.edu.cn (K.W.); shangang.jia@cau.edu.cn (S.J.); caihw@cau.edu.cn (H.C.); maops@cau.edu.cn (P.M.); 2 Department of Agronomy, Purdue University, West Lafayette, IN 47907, USA; yjiang@purdue.edu

**Keywords:** zoysiagrass, rust disease, photosynthesis, RNA-seq, resistance mechanism

## Abstract

Zoysiagrass (*Zoysia japonica*) is a popular turfgrass species and is widely used for sport turf and urban landscape. Zoysiagrass is often infected by *Puccinia zoysiae*, which causes a loss in turf quality. The physiological and molecular mechanisms of rust resistance are poorly understood in this species. In this study, the rust-resistant and susceptible lines of zoysiagrass were inoculated with *P. zoysiae*, and alterations of leaf cell structure, physiological indicators and transcriptomic response were investigated at the various stages of inoculation. After inoculation, the cell membranes, nucleus, mitochondria, and chloroplast were all impaired, followed by abnormal physiological metabolism. The damage occurred earlier and more severely in the susceptible line. Changes in electrolyte leakage and chlorophyll content varied with the genotype and the inoculation stages. The transcriptome analysis showed that plant hormones, MAPK signal transduction pathway, photosynthesis and energy generation pathways were significantly enriched in the early response, in both the resistant and susceptible lines. The results provided insights into the physiological and molecular mechanisms of rust disease resistance and would benefit the breeding of rust-resistant varieties in zoysiagrass and related turfgrass species.

## 1. Introduction

*Zoysia japonica* is a perennial herbaceous plant found in temperate regions with a dense root system, dense and hard leaves, and extensive creeping stolons [1]. It exhibits a strong capacity to develop tillers and is highly adaptive to drought and environments with poor soil fertility. Because of these characteristics, zoysiagrass has become one of the most widely distributed lawn grasses in the world and is often used for sports fields and urban green lawns [2]. However, rust is the most common, long-lasting disease in *Z. japonica* and seriously affects the quality of the lawn. The rust pathogen absorbs nutrients from living plant cells, destroys chlorophyll, and reduces photosynthetic and respiratory rates [3], thus, negatively affecting growth, physiology and overall health of the plants. It has been reported that rust is not only found on zoysiagrass, but it is also commonly found in other warm-season lawns under humid conditions [4]. Severe rust infection can result in the death of the whole area and delay plant regrowth [5], and ultimately, reduce the value of lawns, causing huge economic losses [6,7].

Pathogenic bacteria infection destroys the plant cell structure and causes dysfunction in the organelles, such as the deformation of chloroplasts. Moreover, the loss of function in organelles can affect physiological and biochemical reactions, as well as the expression of related genes related to disease infection [8,9]. Cell walls and organelles act as the first barrier against pathogen infection [8]. In the study of rice basal rot, the internal structure of plant cell organelles was deformed, broken and dissolved with the extension of infection time [10]. The change or rearrangement of chloroplast structure caused by pathogen infection affects its normal function. After being infected by poplar canker disease and wheat stripe rust, cell structure changes were observed, including more deformed chloroplasts and an increased number of swelled and disintegrated mitochondria [11,12]. The disorder of chloroplast composition and function negatively influences photosynthesis and plant defense systems.

To avoid or hinder the infection of pathogens and reduce the incidence of diseases, plants gradually form a series of complex and effective defense response mechanisms [13]. Many plant defense pathways are activated or inhibited by pathogen infection. In general, infection by pathogens can reduce photosynthesis and cause metabolic dysfunction of the plants [14]. Chlorophyll content is a good indicator of the photosynthetic ability of plants under external stress [15]. The disease reduces chlorophyll content to inhibit the normal action of the photosystem. The infection of *Peronospora aestivalis* reduced the chlorophyll content of alfalfa by 28.4% to 68.6%, leading to the yield decline [16]. The foliar disease infection also inhibited the function of photosystem II in alfalfa [17]. At the late stage of infection in wheat stripe rust, the leaves of the infected plants showed significant chlorosis [18] and decreased photosynthetic activity [19,20].

Transcriptome sequencing has been widely used to reveal the response of plant hosts to pathogen infection. A large number of genes and metabolic pathways related to disease resistance have been identified, providing important information for in-depth study on the molecular mechanism of plant–pathogen interaction and disease resistance [8]. For example, transcriptome analysis revealed that Ca^2+^ regulation and phosphorylation pathways were closely related to the response of *Z. japonica* to *R. solani* [21]. In addition, phenylpropanoid biosynthesis, reactive oxygen species, photosynthesis and thiamine metabolism pathways play important roles in wheat resistance to stripe rust [22]. The use of transcriptome data to discover disease-resistant genes is especially desirable and valuable for turfgrass species, given that the genome sequences of many turfgrass types are largely unknown.

To date, the studies on rust disease in zoysiagrass are limited. Our previous works on *Z. japonica* identified the rust-resistant and susceptible materials [23,24], studied leaf tissue structures after infection [6], and investigated the relationship between antioxidant enzyme activity and gene expression and disease resistance [25]. However, the physiological and molecular mechanisms of rust resistance remain largely unclear in zoysiagrass. In this study, we compared changes in cell structure and physiological activity in rust-resistant and susceptible lines. We also performed transcriptome sequencing to explore the metabolic pathways and identify the key genes related to rust resistance in *Z. japonica*. The results will provide insights into mechanisms of rust resistance in zoysiagrass and guide breeding programs for creating disease-resistant varieties.

## 2. Results

### 2.1. Whole-Plant Observations of Z. japonica Leaves after Inoculation

**Figure 1:**
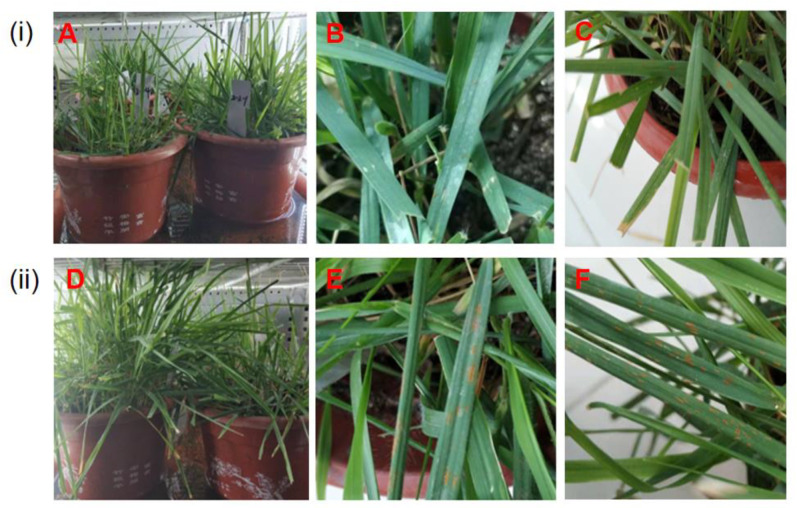
Observation on the leaves of rust-resistant *Z. japonica* (RR) (**A**–**C**) and rust-susceptible *Z. japonica* (RS) (**D**–**F**) after inoculation with *P. zoysiae*. (**i**) Inoculated RR leaves; (**ii**) Inoculated RS leaves; (**A**,**D**) Non-inoculation; (**B**,**E**) 5 days post inoculation (dpi); (**C**,**F**) 15 dpi.

The phenotypic changes in the rust-resistant and rust-susceptible *Z. japonica* leaves inoculated with rust pathogen at 0, 5, and 15 dpi were recorded. As shown in Figure 1i, a few spotty rust spores were found at a few leaf edges and leaf sheaths in rust-resistant *Z. japonica* (RR) at 5 dpi (Figure 1A,B). The leaves of RR remained green, and the leaf epidermis at the affected site had a slight rupture after 15 dpi (Figure 1C). As shown in Figure 1ii, bundle sporangium powder and spore heap of 3–5 mm appeared on the surface of some leaves in rust-susceptible *Z. japonica* (RS) at 5 dpi (Figure 1D,E). The affected site gradually and linearly spread to the whole leaf surface in RS, with observation of an increased number of affected leaves (Figure 1F).

### 2.2. Cellular Observations of Z. japonica Leaves after Inoculation

**Figure 2:**
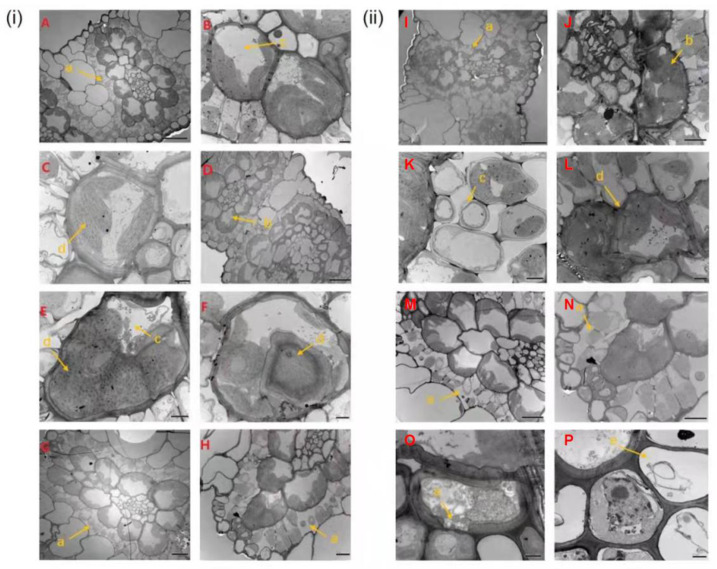
Leaf slices of rust-resistant *Z. japonica* (RR) (**A**–**H**) and rust-susceptible *Z. japonica* (RS) after inoculation with *P. zoysiae*. (**i**) Inoculated RR leaves; (**ii**) Inoculated RS leaves; (**A**,**I**) Non-inoculation; (**B**,**J**) 1 day post inoculation (dpi); (**C**,**K**) 3 dpi; (**D**,**L**) 5 dpi; (**E**,**M**) 8 dpi; (**F**,**N**) 10 dpi; (**G**,**O**) 15 dpi; (**H**,**P**) 20 dpi. (a) mesophyll cell; (b) chloroplast; (c) plasmolysis; (d) cell wall; (e) epidermal cell.

Changes in the cell structure of RR and RS leaves inoculated with rust pathogen were recorded by transmission electron microscope (TEM) at different times. Prior to inoculation, epidermal cells of RR had a complete structure and the mesophyll cells were arranged tightly and neatly (Figure 2A). By 5 dpi, no significant changes were observed in the overall structure in RR (Figure 2B–D). At 8–20 dpi, vacuoles in some vascular bundle sheath cells shrank in RR (Figure 2E–H). For RS, there were few mesophyll cells, larger intercellular space and more loose arrangement in non-inoculated RS compared to that in RR (Figure 2I). By 5 dpi, rust cells were found around the epidermal cells and some mesophyll cells showed separation in the plasma wall in RS (Figure 2J–L). At 8–20 dpi, some mesophyll cells were dissolved into vesicles in RS (Figure 2M–P).

**Figure 3:**
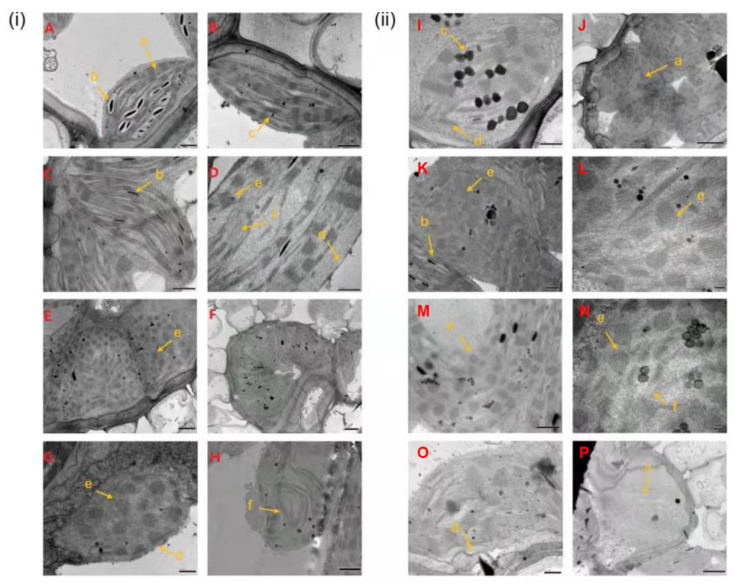
Chloroplast changes of rust-resistant *Z. japonica* (RR) and rust-susceptible *Z. japonica* (RS) after inoculation with *P. zoysiae*. (**i**) Inoculated RR leaves; (**ii**) Inoculated RS leaves; (**A**,**I**) Non-inoculation; (**B**,**J**) 1 day post inoculation (dpi); (**C**,**K**) 3 dpi; (**D**,**L**) 5 dpi; (**E**,**M**) 8 dpi; (**F**,**N**) 10 dpi; (**G**,**O**) 15 dpi; (**H**,**P**) 20 dpi. (a) chloroplast; (b) starch granule; (c) plastid globule; (d) chloroplast membrane; (e) granule; (f) matrix thylakoid.

Before inoculation, chloroplasts of RR in RS were mostly flat and elliptic, and distributed near the plasma membrane of the cells (Figure 3A). At 1 dpi, starch grains in chloroplast stroma disappeared and reappeared at 3 dpi in RR (Figure 3B,C). At 5–15 dpi, compared with that of non-inoculation, the amount of starch grains synthesized in chloroplast was reduced and some cells had enlarged chloroplast morphology, smaller internal grana area, and fuzzy thylakoid structure (Figure 3D–G). At 20 dpi, the plasmid lamella of chloroplast was distorted and fractured in RR (Figure 3H). Before inoculation, the structure of chloroplast in RS was clear and complete (Figure 3I). At 1–8 dpi, the chloroplasts of a few vascular bundle sheath cells were swollen and the chloroplast structure was deformed in RS (Figure 3J–N). At 10–20 dpi, the synthesis of starch grains in the chloroplasts of RS was further reduced, with some ruptured chloroplasts, and cavities appeared (Figure 3O,P).

**Figure 4:**
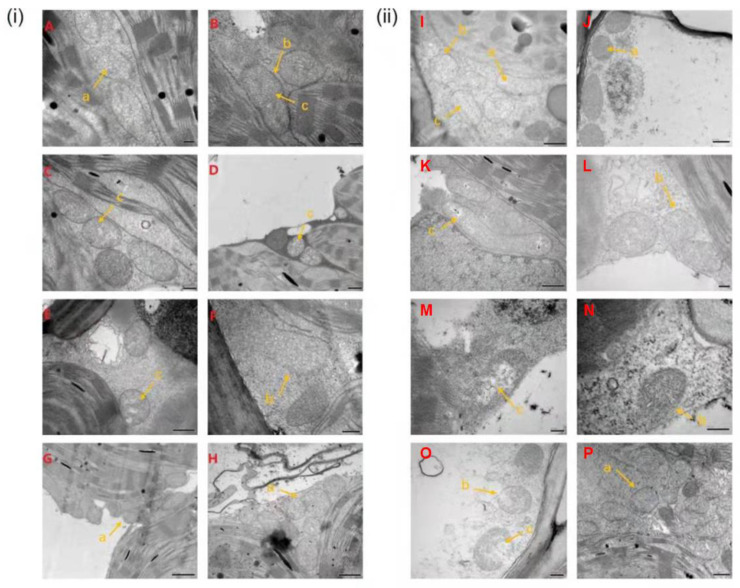
Mitochondrial changes in rust-resistant *Z. japonica* (RR) and rust-susceptible *Z. japonica* (RS) after inoculation with *P. zoysiae*. (**i**) Inoculated RR leaves; (**ii**) Inoculated h RS leaves; (**A**,**I**) Non-inoculation; (**B**,**J**) 1 day post inoculation (dpi); (**C**,**K**) 3 dpi; (**D**,**L**) 5 dpi; (**E**,**M**) 8 dpi; (**F**,**N**) 10 dpi; (**G**,**O**) 15 dpi; (**H**,**P**) 20 dpi. (a) mitochondria; (b) mitochondrial membrane; (c) cristae.

Before inoculation, the mitochondria of the internal cells in the leaves of RR were distributed in the cell matrix in the form of ovate, with a double-layer membrane structure (Figure 4A). By 5 dpi, there was no significant change in the mitochondrial structure of RR (Figure 4B–D). At 8–20 dpi, a large number of mitochondria in some vascular bundle sheath cells clustered together and the number of mitochondria tended to increase in RR (Figure 4E–H). For non-inoculated RS, there was a clear internal crest structure (Figure 4I). By 5 dpi, cavities were observed in some mitochondrial matrices and double-layer membrane structure folded deformations in the leaves of RS (Figure 4J–L). At 8–20 dpi, partial mitochondria were completely disintegrated and the number of mitochondria increased in RS (Figure 4M–P).

**Figure 5:**
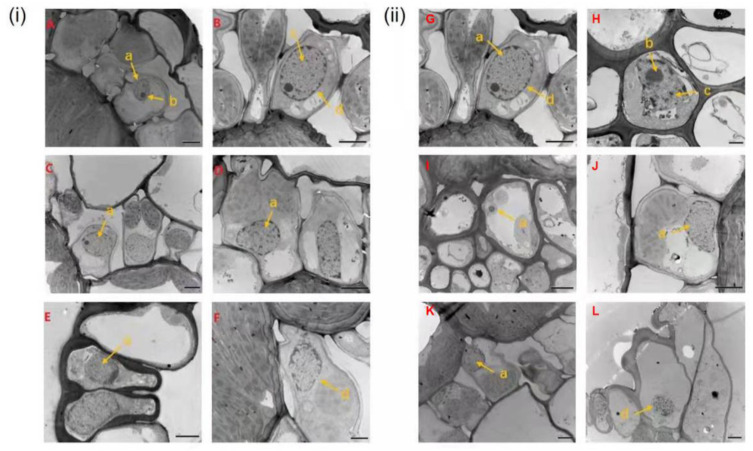
Changes in the nuclei of rust-resistant *Z. japonica* (RR) and rust-susceptible *Z. japonica* (RS) after inoculation with *P. zoysiae*. (**i**) Inoculated RR leaves; (**ii**) Inoculated RS leaves; (**A**,**G**) Non-inoculation; (**B**,**H**) 5 day post inoculation (dpi); (**C**,**I**) 8 dpi; (**D**,**J**) 10 dpi; (**E**,**K**) 15 dpi; (**F**,**L**) 20 dpi. (a) nucleus; (b) nucleolus; (c) nuclear matrix; (d) nuclear membrane.

At 0–5 dpi, the nuclei of the mesophyll cells were spherical and the double-layer membrane structure was clear and large in RR (Figure 5A,B). At 8–10 dpi, nuclear nucleolus in mesophyll cells become smaller in RR (Figure 5C,D). At 15–20 dpi, the nucleolus completely dissolved and nuclear membrane structure was blurred (Figure 5E,F). At 0–5 dpi, the nuclei contained spherical nucleoli (Figure 5G,H). For RS at 5–10 dpi, the nucleolus in the mesophyll nucleus was dissolved with ruptured nuclear membrane, and the nuclear matrix leaked (Figure 5I,J). At 15–20 dpi, more epidermal and mesophyll cells showed a decreased number of nucleolus, and finally the nuclear structure was disintegrated in RS (Figure 5K,L).

### 2.3. Electrolyte Leakage (EL) and Chlorophyll Content (Chl)

**Figure 6:**
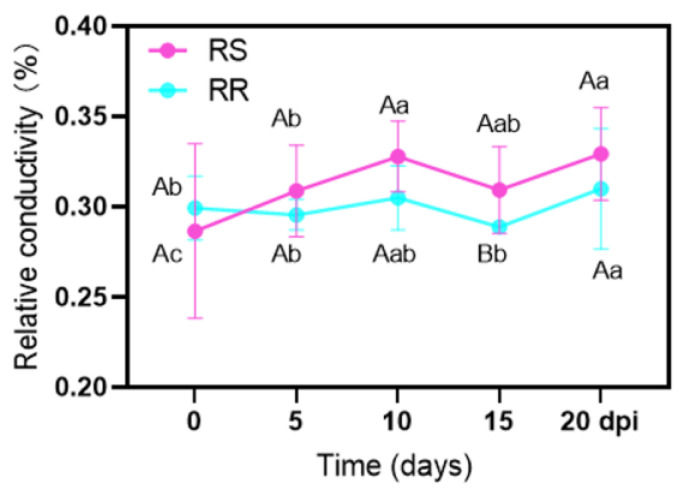
Changes in electrolyte leakage in rust-resistant *Z. japonica* (RR) and rust-susceptible *Z. japonica* (RS) after inoculation with *P. zoysiae*. Different lowercase letters mean significant difference among different days in the same material at *p* < 0.05. Different capital letters indicate significant difference between the two materials at a given date at *p* < 0.05.

With the extension of inoculation time, the change in leaf EL of RR was relatively small, at 0–15 dpi (Figure 6). Compared with non-inoculation, EL increased significantly at 20 dpi in RR, reaching 34.2%. The EL of RS was 34.7% at 10 dpi and 20 dpi, which was significantly higher than that at 0 and 5 dpi. Before inoculation, EL did not differ between RR and RS, while it was significantly higher in RS than in RR at 15 dpi.

**Figure 7:**
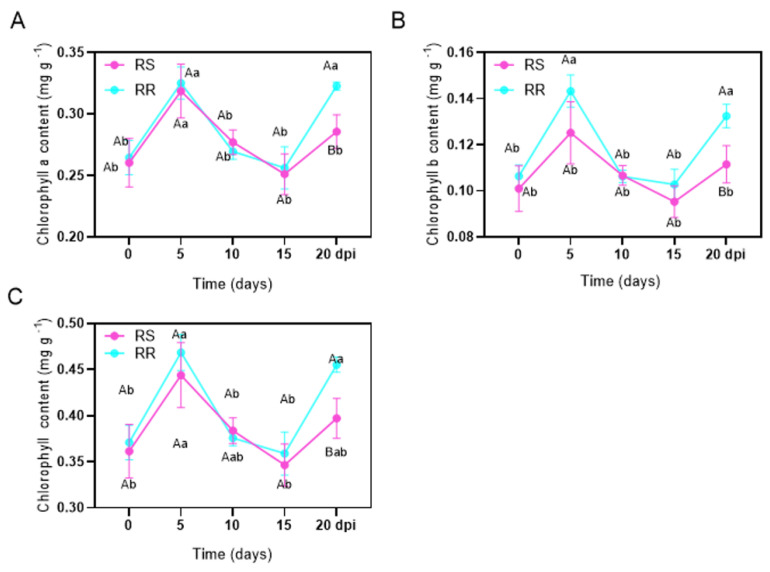
Change in chlorophyll content in rust-resistant *Z. japonica* (RR) and rust-susceptible *Z. japonica* (RS) after inoculation with *P. zoysiae* (**A**–**C**). Different lowercase letters mean significant difference among different days in the same material at *p* < 0.05. Different capital letters indicate significant difference between the two materials at a given date at *p* < 0.05.

The contents of Chl a, Chl b and total Chl (a + b) increased first, then decreased and increased again, in both RR and RS after inoculation (Figure 7). Compared to non-inoculation, the contents of Chl a, Chl b and total Chl in RR increased significantly at 5 dpi, while the contents of Chl b in RS did not change. At 10–15 dpi, the contents of Chl a, Chl b, and total Chl did not alter significantly compared to those before inoculation in RR and RS (Figure 7). At 20 dpi, the contents of Chl a, Chl b and total Chl in RR increased significantly, while no differences in these Chl parameters were found between inoculated and non-inoculated RS (Figure 7). At 5 dpi and 20 dpi, the contents of Chl a, Chl b and total Chl (a + b) in RR were significantly higher than that in RS.

### 2.4. Transcriptome Analysis

**Figure 8:**
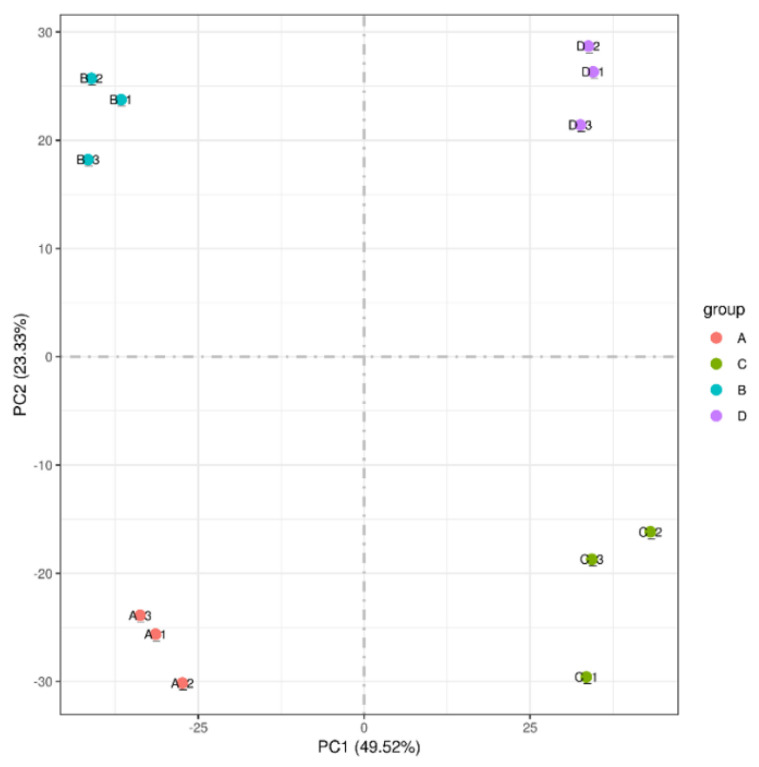
Principal component analysis between transcriptome samples. **A**, RR without inoculation; **B**, RS without inoculation; **C**, RR inoculated for 5 days; **D**, RS inoculated for 5 days.

Analysis of the transcriptome sequencing in leaves was carried out for comparisons between RR and RS, without inoculation and at 5 dpi of inoculation. The results of the principal component analysis showed that samples were separated into four groups (Figure 8).

The pairwise comparisons of DEGs were conducted among the four groups (Table 1). Before inoculation, there were 5371 DEGs in RR1 vs. RS1, including 2709 up-regulated and 2662 down-regulated DEGs. After inoculation, there were 1427 up-regulated and 1747 down-regulated DEGs in RR1 vs. RS2. A total of 11,234 DEGs were identified in RR1 vs. RS2, including 5382 up-regulated and 5852 down-regulated. There were 11,957 DEGs in RS1 vs. RS2, including 5900 up-regulated and 6057 down-regulated (Table 1).

**Figure 9:**
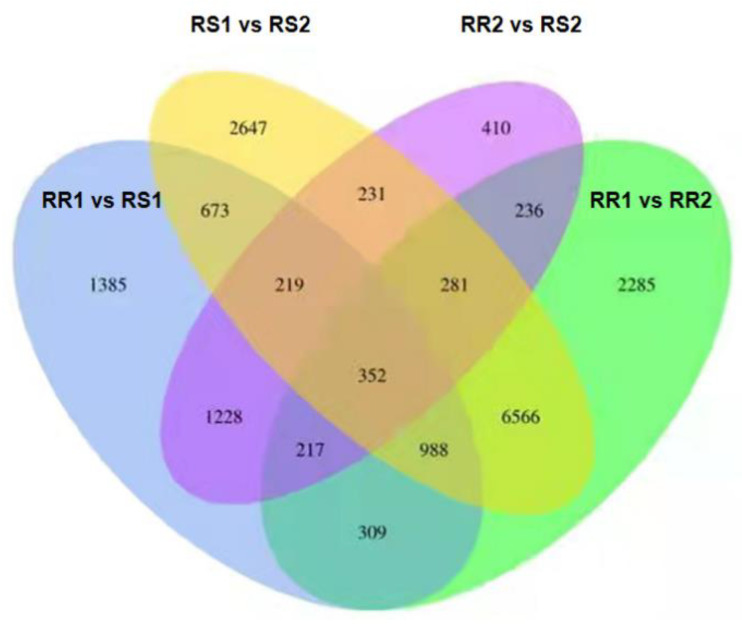
Venn diagram of differentially expressed genes in rust-resistant (RR) and rust-susceptible (RS) *Z. japonica* lines. RR1, RR without inoculation; RS1, RS without inoculation; RR2, RR inoculated for 5 days; RS2, RS inoculated for 5 days.

The unique DEGs in each pairwise comparison were 1385 (RR1 vs. RS1), 2647 (RS1 vs. RS2), 410 (RR2 vs. RS2) and 2285 (RR1 vs. RS2) (Figure 9). The DEGs of RR and RS before inoculation and after inoculation were compared, i.e., RR1 vs. RS1 and RR2 vs. RS2. There were 4513 DEGs of RR2 vs. RS2 relative to RR1 vs. RS1. The DEGs before and after inoculation of RS were compared with those before and after the inoculation of RR (RR1 vs. RR2 vs. RS1 vs. RS2), and there were 12,965 DEGs of RR1 vs. RR2, relative to RS1 vs. RS2.

**Figure 10:**
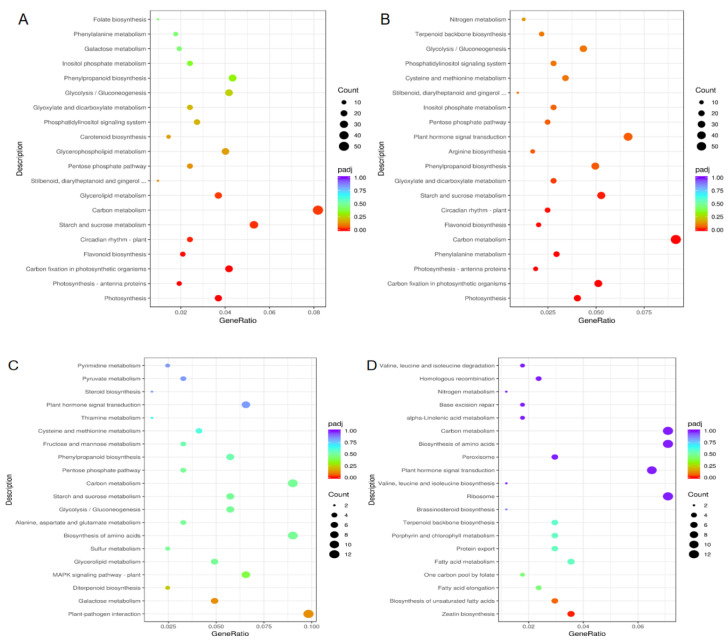
KEGG enrichment analysis of differentially expressed genes (DEGs) in rust-resistant (RR) and rust-susceptible (RS) *Z. japonica* lines. RR1, RR without inoculation; RS1, RS without inoculation; RR2, RR inoculated for 5 days; RS2, RS inoculated for 5 days. (**A**) Up-regulated gene RR2 relative to RR1; (**B**) Up-regulated gene RS2 relative to RS1; (**C**) Up-regulated genes of RR2 relative to RS2; (**D**) Down-regulated genes of RR2 relative to RS2.

KEGG enrichment analysis on DEGs revealed a difference in the metabolism pathways between RR and RS (Figure 10). Compared with the non-inoculated RR (RR1), the enriched pathways of up-regulated DEGs were related to photosynthesis, carbon metabolism, carbon fixation in photosynthetic organisms, and photosynthesis-antenna protein in RR at 5 dpi (RR2) (Figure 10A). As for RS2 vs. RS1, the photosynthesis pathway was up-regulated. In addition, the up-regulated enrichment pathways of RS at 5 dpi (RS2) were involved in plant hormone signal transduction, phosphatidylinositol signal, and steroid and curcumin biosynthesis, compared with those of non-inoculated RS (RS1) (Figure 10B). Compared with RS at 5 dpi (RS2), the enriched pathways of up-regulated DEGs were mainly related to plant–pathogen interaction and galactose metabolism (Figure 10C), while the enriched down-regulated pathways were zeatin biosynthesis and biosynthesis of unsaturated fatty acids pathways in RR2 vs. RS2 (Figure 10D).

**Figure 11:**
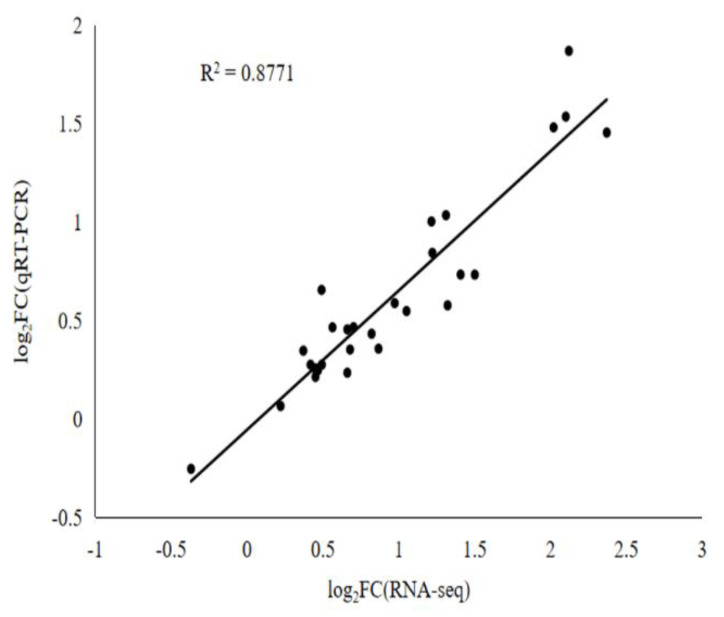
The correlation between the results of RNA-seq and qRT-PCR.

To verify the transcriptomic sequencing results, 14 differentially expressed genes (DEGs) were randomly selected for qRT-PCR analysis. The results showed that the expression of these genes in qRT-PCR was strongly consistent (R^2^ = 0.8771) with those in the transcriptomic analysis (Figure 11).

## 3. Discussion

### 3.1. Leaf Cell Structure and Physiological Changes after Inoculation with Rust Pathogen

Mitochondria are the main organelles controlling ROS homeostasis in the cytoplasm, and dynamic changes in the morphology of mitochondria may indicate a stress injury [26]. In this study, the mitochondria of RR and RS leaves inoculated with rust pathogen showed that the mitochondrial structure of RS had already changed at 3 dpi, and the degree of damage was greater at the later stages. It is worth noting that RR did not appear as abnormal mitochondria until 8–15 dpi (Figure 4), suggesting that RR has a stronger capacity in resisting rust pathogen infection than RS. Studies on fungal diseases demonstrated that increasing respiration was a basic response to injury in most infected host tissues [27]. The increase and aggregation of mitochondria were observed in RR and RS at a later stage of infection, which might be due to the enhanced respiration at the infected site caused by pathogen infection.

The cell membrane system is damaged under the stress of pathogenic bacteria [28]. In this study, the change in the electrolyte leakage was minor in RR after inoculation, compared with RR with no inoculation; however, the electrolyte leakage of RS increased significantly at 10 dpi and 20 dpi (Figure 6). Therefore, infection of *P. zoysiae* could lead to increased cell membrane damage of the zoysiagrass leaves and break the integrity of the mesophyll cell membrane, especially in the susceptible line. Compared with RS, less damage was found in RR, indicating that RR had better resistance to the rust of *P. zoysiae*.

Previous studies have shown that even minor damage to photosynthesis can weaken the defense systems and increase the susceptibility of the plants to pests and pathogens [27]. The chloroplast disorder led to the change or rearrangement of the chloroplast ultrastructure, which could be related to the development of plant symptoms after pathogen infection [29]. Our results indicated that different degrees in malformation of the chloroplast structure occurred with the extension of infection time. Grana, grana lamella and other structures in leaf cells of the RS leaves were dissolved, and thylakoid membrane was destroyed earlier than RR. This could decrease the photosynthetic efficiency of the plants, accelerating the infection process of pathogenic bacteria (Figure 3). Studies on downy mildew and powdery mildew showed that the chlorophyll content in leaves was positively correlated with the resistance of plants [30]. The chlorophyll content in the RR at 5 and 20 dpi significantly increased compared to non-inoculated plants, but was significantly higher than RS at 5 and 20 dpi (Figure 7). The results indicated that more severe damage in chloroplasts in RS caused a decline in chlorophyll biosynthesis and resulted in low photosynthetic capacity, ultimately leading to lower resistance to pathogens than RR. Our results supported that the chlorophyll content was positively correlated with the rust resistance of *Z. japonica*.

### 3.2. Transcriptomic Response to Inoculation with Rust Pathogen

#### 3.2.1. Photosynthesis Was Closely Related to the Rust Resistance of *Z. japonica*

Comparing RR2 with RR1 and RS2 with RS1, the genes responding to the *P. zoysiae* infection (multiple changes in the top ten) were identified, including those encoding alkaline leucine zipper (basic region leucine zipper motif, bZIP) transcription factor, Dirigent-like protein and cytochrome P450, glycosyl hydrolase family, chlorophyll-binding protein, light system IReaction center subunit, EamA auxin transporter family, etc. (Table S2). After inoculation, the photosynthetic system, chlorophyll metabolism pathway, bZIP transcription factors and transporters were closely related to pathogen infection, indicating a role of these pathways and genes in plant responses to pathogen. Comparing RS1 with RR1, the genes responding to the *P. zoysiae* infection (multiple changes in the top ten) were identified, including those encoding plant antimicrobial peptide alkaline and eukaryotic glutathione synthase, indicating that the RR1 expressed more antimicrobial peptides on genetic factors to enhance rust resistance. Comparing RS2 with RR2, glucose transporter and antifungal-related genes were significantly up-regulated in the glucose metabolism pathway, indicating that rust-resistant material at 5 dpi (RR2) could synthesize more sugars, energy and anti-fungal-related secondary metabolites to resist the stress of pathogens compared to rust-susceptible material at 5 dpi (RS2). Light harvesting complex (LHC) proteins are located on PS I and PS II. In this study, genes encoding subunit proteins Lhca1, Lhca3, Lhca5, Lhcb1, Lhcb4, Lhcb5, and Lhcb7 on light harvesting complex were significantly up-regulated after inoculation in RR and RS (Table S2), suggesting that the light capture ability and the photosynthetic efficiency of zoysiagrass plants were improved to adapt to the invasion of pathogens. In addition, the up-regulation of multiple genes in the light harvesting complex was generally higher in RS than that in RR, which was consistent with a greater chloroplast disruption in RS observed by TEM.

The infection of pathogens can cause chlorosis in plants, but the molecular mechanism is unclear [31]. In our study, the leaves of RR remained green at 15 dpi, while the leaves of RS slightly faded at the infected sites at 5 dpi (Figure 1). In the porphyrin and chlorophyll metabolism pathways, genes encoding chlorophyll a oxygenase, magnesium-proporphyrin IX monomethyl ester, heme oxygenase (HO), magnesium chelatase subunit D, chlorophyll b reductase, and chlorophyll enzyme were significantly up-regulated in both RR and RS after inoculation (Table S2). Previous studies have shown that magnesium-chelating enzyme and protoporphyrin methyl transferase (ChlM) were the key enzymes of chlorophyll biosynthesis [32]. ChlM influenced the formation of the PS I and PS II, regulated magnesium-chelating enzyme activity, and played a key role in maintaining the proper progress of photosynthesis [33]. Our results demonstrated that the increased chlorophyll content of RR and RS leaves at 0–5 dpi was consistent with up-regulation of chlorophyll biosynthetic genes. However, the photosynthetic components and chlorophyll-biosynthesis-related genes were significantly down-regulated after pathogen infection in *Phytophthora sojae* [34,35]. Chlorophyll b reductase and chlorophyllase are recognized as chlorophyll-degrading enzymes [31]. The increased expressions of chlorophyll b reductase and chlorophyllase genes after inoculation in RR and RS could contribute to decreases in chlorophyll. In the studies on wheat stripe rust, the early chlorosis was associated with the WKS1.1-mediated promotion of phosphorylation in PsbO, one of the three external protein subunits (PsbO, PsbP, and PsbQ) of the photosystem II (PSII) supercomplex [18,32,36]. Compared with the non-inoculated materials, we found that the gene expressions of 13 genes, encoding PsbO, PsbP, Psb27, PsaD, and PsaE protein in the photosynthetic system, Fd protein, and the related genes involved in ATP synthesis, increased in both RR and RS at 5 dpi, while the expressions of genes encoding PsbQ and PetJ were significantly lower (Table S2). We speculated that the subunit proteins on thylakoid membrane proteins were involved in regulating the response of zoysiagrass after being infected by *P. zoysiae*. However, the molecular mechanisms need to be further clarified.

#### 3.2.2. Other Defense Systems Closely Related to the Response of *Z. japonica* to Pathogen Infection

Previous studies have shown that bZIP transcription factors are mainly located in the nucleus and involved in light signals, plant hormones, injury, pathogen defense and response to various environmental stresses [37]. Proteins encoded by the Dirigent-like gene (DIR) are involved in lignin formation and plant pathogen defense [38]. Comparing RR1 and RS1, the top ten genes were genes encoding plant antimicrobial peptides and eukaryotic glutathione synthase, indicating that more antimicrobial peptides were expressed in the genetic factors of RR1 to enhance its disease resistance. Compared with RS2, sugar-transporter-associated genes and antifungal genes increased in RR2. It suggested that RR synthesized more sugars, energy and anti-fungal-related secondary metabolites for rust resistance.

After inoculation, up-regulated genes that jointly responded to pathogen infection were excluded from RR and RS (RR2 vs. RR1 vs. RS2 vs. RS1). The up-regulated unique top ten genes in RR were transfer of enzymes, diphenylethylene synthetase, sugar-transporter protein, DNA binding domain, etc. Stilbene synthase is involved in the synthesis of phytoprotectin, and phytoprotectors are a kind of protective compound, produced when plants are invaded by pathogenic microorganisms. Previous studies showed that transferring styrene synthase into rice improved the disease resistance of plant blast resistance [39]. After inoculation, the gene-coding amylase, zinc finger protein transcription family and reverse transcriptional transposin in the unique top ten genes were up-regulated in RS. Amylase, as a non-specific enzyme, can degrade chitosan, a component in the fungal cell wall that alleviates the infection of pathogenic bacteria on the host [40]. In *Arabidopsis thaliana* and tobacco, genes encoding zinc finger protein family were expressed in guard cells, which might be involved in such defense reactions as the inducement of stomatal closure by promoting pathogen resistance [41].

## 4. Materials and Methods

### 4.1. Plant Materials and Growth Conditions

#### 4.1.1. Materials for Inoculation

Through the preliminary screen of zoysiagrass germplasm materials for rust resistance in the field for multiple years [23,24,42], the rust-resistant (RR) and rust-susceptible (RS) lines were identified and used for this experiment. The RR and RS materials were originally obtained from the propagation of a single seed and were grown in greenhouse for 3~4 years, with stable phenotypes for rust response. The rust fungi strain used for inoculation was determined as *P. zoysiae*, based on morphological and molecular identifications by Zhang [25]. The strains were cultured in a climate chamber with temperatures of 15 °C to 25 °C, 90% humidity, and an 8 h photoperiod of 600 µmol·m^−2^ s^−1^.

#### 4.1.2. Methods for Inoculation

For each *Z. japonica* line, four tillers were selected from one single plant and transplanted into small pots (18 cm diameter, 25 cm height) and grown in a greenhouse for 60 days with temperatures of 25 °C to 30 °C, 70% humidity, an 8 h photoperiod of 800 µmol·m^−2^ s^−1^. Plants were healthy and uniform with good vigor prior to inoculation. *P. zoysiae* spores were collected and used to prepare a spore suspension as pathogen donor. The concentration of spore suspension was adjusted to 0.3 mg/mL (containing 0.2% gelatin) and was sprayed on the plant leaves for inoculation according to a previous method described by Nyassé et al. [43]. After inoculation, pots were covered with plastic bags, filled with air, and closed with rubber bands. Pots were placed in an illuminating incubator for 24 h at 28~30 °C and then in a moist chamber for 2 to 3 days.

### 4.2. Observations of the Inoculated Leaves

Leaf samples collected at 0, 1, 3, 5, 8, 10, 15, and 20 days post inoculation (dpi) of the RR and RS lines were used for transmission electron microscope (TEM) observation with four replicates. The tissue samples (3–5 mm) from the middle of leaves were put into the fixing solution for 2 min at room temperature in the dark for 48 h, and then stored at 4 °C refrigerator. After fixation with 2.5% glutaraldehyde and dehydration with 50% ethanol and embedding with pure acetone, the samples were placed in an oven at 37 °C overnight. Samples were sliced to 50–60 nm with ultra-thin slicer, then stained and coated into slices. The upper surface of the leaves was observed and photographed under the field of vision of a JEM-1230 TEM (NSK Ltd., Tokyo, Japan).

### 4.3. Determination of Electrolyte Leakage

Approximately 0.2 g of RR and RS leaves were taken at 0, 5, 10, 15 and 20 dpi for measuring electrolyte leakage. The leaves were cut into 3–5 mm, soaked in tubes containing 20 mL ultra-pure water, and shaken for 16 h. The initial conductivity R1 was measured with a conductivity meter (UH-5300), and conductivity R2 was measured after the tissues were autoclaved for 30 min and cooled to room temperature. The electrolyte leakage was calculated by R = (R1/R2) × 100%.

### 4.4. Chlorophyll Determination

Approximately 50 mg of leaves at 0, 5, 10, 15, and 20 dpi of RR and RS plants were cut into 3–5 mm and were soaked in 95% ethanol for 48 h. Next, 1 mL of the extract was used for recording absorbance at 665 nm, 649 nm, and 470 nm in a UV spectrophotometer. The chlorophyll content was calculated based on the following formula [44].

Chlorophyll a content = 13.95 × OD665 − 6.88 × OD649;       

Chlorophyll b content = 24.96 × OD649 − 7.32 × OD665;       

Chlorophyll a + b content = (1000 × OD470 − 2.05 × Ca − 114.8 × b)/245. 

### 4.5. Transcriptome Sequencing and Analysis

We performed transcriptome sequencing for the four treatments, i.e., RR without inoculation (RR1), RR with inoculation at 5 dpi (RR2), RS without inoculation (RS1), and RS with inoculation at 5 dpi (RS2), with three replicates for a total of 12 samples. RNA extraction and transcriptome sequencing were performed by Beijing Novogene Co., Ltd., Beijing, China. Briefly, RNA quality was assessed for RNA integrity and DNA contamination by agarose gel electrophoresis. More than 1 μg of total RNA was prepared for library construction by using the NEBNext^®^ UltraTM RNA Library Prep Kit (Illumina, NEB, Ipswich, MA, USA). The insert size was evaluated with Agilent 2100 bioanalyzer. Low-quality reads were removed, and clean data was subject to quality evaluation of Q20, Q30, and GC content. HISAT2 v2.0.5 was used to align the clean reads to the Zoysia genome. The differentially expressed genes (DEGs) were identified by the R package DESeq2 (Love et al., 2014), according to fold change ≥2.0 and the adjusted *p* value ≤ 0.05. GO and KEGG enrichment analyses were performed in R package clusterProfiler [45]. The RNA-seq raw data were deposited in the Short Reads Archive (SRA) of NCBI (accession No: PRJNA824948).

### 4.6. Quantitative RT-PCR Analysis

All gene-specific primers used in qRT-PCR experiments were designed by using Primer Premier 5 (Table S1). The RNA was extracted from each sample and reverse transcribed into cDNA by using the PrimeScriptTMRT reagent kit (RR047A, TAKARA, Japan). cDNA was diluted to 10-fold for qRT-PCR analysis. Each sample was amplified three times using SYBR Premix Ex Taq (Takara, Japan) on the Bio-Rad CFX96 real-time PCR detection system (Bio-rad, Hercules, CA, USA), with *ZjActin* as the internal control (Table S1). The relative quantification (2^−∆∆CT^) of target gene expression was calculated according to the method described by Schmittgen et al. [46].

### 4.7. Statistical Analysis

The data of electrolyte leakage and chlorophyll content were analyzed using SPSS 18.0 (SPSS. Inc., Chicago, IL, USA). Pairwise comparison was conducted from different treatments with three replicates. Differences were deemed significant when *p* < 0.05.

## 5. Conclusions

**Figure 12:**
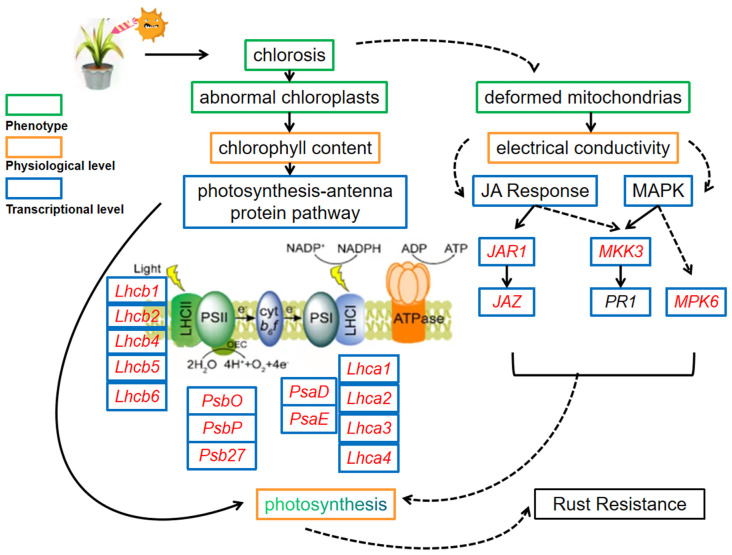
A molecular regulatory scheme of *Z. japonica* in response to *P. zoysiae.* Red letters indicate the up-regulated genes. Dotted lines indicate direct or indirect effect.

Based on the above results, we mapped a molecular regulatory scheme of response for *Z. japonica* after infection by *P. zoysiae* (Figure 12). Rust pathology *P. zoysiae* destroyed the epidermis structure of zoysiagrass leaves and damaged the internal chloroplasts, mitochondria, nuclei, and other organelles, especially in the susceptible line. Electrolyte leakage and chlorophyll content varied with the genotype and the inoculation stages. Transcriptome sequencing analysis revealed that photosynthesis, chlorophyll metabolism, plant hormones, MAPK signal transduction pathway and secondary metabolite pathways were enriched among the top ten up-regulated gene pathways after the inoculation. We speculate that in the early stage of response to pathogen infection, *Z. japonica* mainly generated more organic matter, energy, active signal transduction pathways and synthesis of secondary metabolites, through improving photosynthetic efficiency to resist pathogen invasion. Photosynthesis plays an important role in the resistance of zoysiagrass to rust disease. The key network and mechanism of rust resistance in *Z. japonica* through photosynthesis need to be further studied. These results provided a foundation for a better understanding of the molecular mechanisms of rust resistance in zoysiagrass and also established a theoretical basis for breeding programs for creating disease-resistant varieties.

## Figures and Tables

**Table 1:** Statistics of different genes.

Combinations	All	Up	Down	Threshold
RR1 vs. RS1	5371	2709	2662	DESeq2 padj < 0.05
RR1 vs. RR2	11,234	5382	5852	DESeq2 padj < 0.05
RS1 vs. RS2	11,957	5900	6057	DESeq2 padj < 0.05
RR2 vs. RS2	3174	1427	1747	DESeq2 padj < 0.05

Note: RR1, non-inoculated rust-resistant *Z. japonica* (RR); RS1, non-inoculated rust-susceptible *Z. japonica* (RS); RR2, rust-resistant *Z. japonica* (RR) inoculated for 5 days; RS2, rust-susceptible *Z. japonica* (RS) inoculated for 5 days.

## Data Availability

The datasets used and/or analyzed during the current study are available from the corresponding author on reasonable request.

## Supplementary Materials

The following are available online at https://www.mdpi.com/article/10.3390/ijms23084185/s1.
