# Supplementary material for: Physiological and Molecular Responses of Zoysia japonica to Rust Infection
Source: Int J Mol Sci. 2022 Apr 10;23(8):4185. doi: 10.3390/ijms23084185 (PMC9026483; doi:10.3390/ijms23084185)
Supplement: Supplementary file 1 [file ijms-23-04185-s001.zip › ijms-1650318-supplementary.pdf]

Supplemental Table S1. Primer sequences of RT-qPCR

Gene	Forward primer (5'-3')	Reverse primer (5'-3')
Zjn_sc00061.1.g01070.1.sm.mkhc	ATGTCCTGCTCTTTCACTAACC	AGGTGCTTGGGAAGAACATAC
Zjn_sc00007.1.g00600.1.am.mkhc	CTTGCGTTGCTAGGTGTCT	GATCCTCCTCTCATCACCAATAAC
Zjn_sc00014.1.g01250.1.sm.mkhc	CATCCCTGACTTGTTGAGTGT	CCAGTCTCTTCTTGTGTCCTTATC
Zjn_sc00026.1.g00890.1.sm.mk	TCAAGTCCACGATGCAGATATAC	GCGGCTGCTGTAGATGAA
Zjn_sc00015.1.g01220.1.sm.mkhc	GACCTCTGTGCCTCTTGTATC	CGGACCTGAACTGTCATTGT
Zjn_sc00009.1.g10660.1.sm.mkhc	CAGGGCCAAGAGTCGAATAAA	CAATAGAGGTGAGGTGACAGAATC
Zjn_sc00065.1.g00070.1.am.mkhc	GACTGCCGCAAGAAGTTAGA	CCTCTGAGATGGACGACTTTG
Zjn_sc00011.1.g07430.1.sm.mkhc	GAAGCCGTCTCCGAAAGATAG	CCAGCCATTGCAAGAGAAAC
Zjn_sc00034.1.g01350.1.am.mk	TCACGCATATCGAGCCTAAC	CCGCAGGATTGTTGCTATTG
Zjn_sc00112.1.g00080.1.sm.mkhc	CTTACATCCAGCAGCTAGAGAC	AGTGACCCGGAGGATACA
Zjn_sc00043.1.g03070.1.am.mkhc	AGCTGAGGATGCTCTTTCAC	GTTTGCCACATTCCCTGTTG
Zjn_sc00086.1.g01920.1.am.mkhc	ATACGACGACATGGCAGAAC	GGAAGGGAAGATGTTCAGAGTC
Zjn_sc00046.1.g00130.1.am.mkhc	ATGTTAGTTGGTGCATATC	GTGGACATAATCTGGAATAG
Zjn_sc00085.1.g00510.1.sm.mkhc	TCTCAAGGGTGAGGCTAAGA	TACGTGACAGTGCCTACAAAG
ZjActin	GCTCAGTCCAAGAGAGGTATTC	TGATGCCAGATCTTCTCCATATC

Supplemental Table S2. Variation multiples of the TOP 10 differentially expressed genes

Gene ID	Fold change	Description
RR2 vs RS1		
Zjn_sc00105.1.g01770.1.sm.mkhc	10.23896141	PF00170:bZIP transcription factor|PF12498:Basic leucine-zipper C terminal
Zjn_sc00079.1.g00420.1.sm.mk	9.69963256	PF03018:Dirigent-like protein
Zjn_sc00045.1.g00440.1.am.mk	9.631664587	PF00067:Cytochrome P450
Zjn_sc00061.1.g00840.1.am.mkhc	8.677039724	PF00704:Glycosyl hydrolases family 18
Zjn_sc00136.1.g00270.1.am.mkhc	8.591381602	Cytochrome P450
Zjn_sc00036.1.g03030.1.sm.mk	8.533376139	Chlorophyll A-B binding protein
Zjn_sc00029.1.g05340.1.sm.mkhc	8.533270983	Profilin
Zjn_sc00019.1.g00820.1.am.mk	8.15452055	Chlorophyll A-B binding protein
Zjn_sc00004.1.g01410.1.sm.mk	8.084498851	Photosystem I reaction centre subunit III
Zjn_sc00009.1.g06990.1.am.mk	8.000347921	Multicopper oxidase|PF07731:Multicopper oxidase|PF07732:Multicopper oxidase
RR1 vs RS1		
Zjn_sc00080.1.g00140.1.am.mk	10.43880199	Plant antimicrobial peptide
novel.209	10.04004609	-
novel.470	9.998686012	-
Zjn_sc00029.1.g00490.1.sm.mk	9.609114204	-
Zjn_sc00048.1.g00650.1.sm.mkhc	9.510304804	Eukaryotic glutathione synthase, ATP binding domain|PF03199:Eukaryotic glutathione synthase
Zjn_sc06150.1.g00010.1.am.mk	9.27628232	-
novel.860	9.199508511	-
novel.894	9.161751983	-
novel.509	9.158733433	-
Zjn_sc00021.1.g02260.1.am.mk	8.973213948	-
RR2 vs RS2		
Zjn_sc00004.1.g08640.1.sm.mk	26.76601782	Late embryogenesis abundant (LEA) group 1
novel.860	11.81139662	-

Zjn_sc00050.1.g03350.1.sm.mk	11.68672099	-
Zjn_sc00004.1.g04210.1.am.mk	10.38771445	Sugar (and other) transporter|PF05042:Caleosin related protein
Zjn_sc00010.1.g03310.1.am.mk	10.26852814	-
novel.209	10.18418016	-
Zjn_sc00735.1.g00010.1.am.mk	10.08503895	Salt stress response/antifungal
novel.205	10.07338932	-
Zjn_sc00006.1.g04150.1.sm.mkhc	10.04673758	-
Zjn_sc00048.1.g00650.1.sm.mkhc	10.04554595	Eukaryotic glutathione synthase, ATP binding domain|PF03199:Eukaryotic glutathione synthase
RS2 vs RS1		
Zjn_sc00112.1.g00250.1.sm.mk	10.58316139	NOP5NT (NUC127) domain
Zjn_sc00027.1.g01530.1.sm.mk	8.614229308	Chlorophyll A-B binding protein
Zjn_sc00045.1.g00440.1.am.mk	8.538952384	Cytochrome P450
Zjn_sc00105.1.g01770.1.sm.mkhc	8.431686706	bZIP transcription factor|PF12498:Basic leucine-zipper C terminal
Zjn_sc00036.1.g03030.1.sm.mk	7.766097114	Chlorophyll A-B binding protein
Zjn_sc00019.1.g00820.1.am.mk	7.688367413	Chlorophyll A-B binding protein
Zjn_sc00004.1.g00540.1.sm.mkhc	7.672365572	EamA-like transporter family
Zjn_sc00037.1.g00930.1.am.mk	7.377783225	Chlorophyll A-B binding protein
Zjn_sc00066.1.g01570.1.am.mk	7.375853669	-
Zjn_sc00001.1.g06810.1.sm.mkhc	7.366299515	Chlorophyll A-B binding protein
RR2 vs RR1 v RS2vs RS1<br>RR2 vs RR2		
Zjn_sc00054.1.g00460.1.am.mk	7.331675014	Protein of unknown function (DUF3339)
Zjn_sc00103.1.g00660.1.am.mk	7.017735734	Sugar (and other) transporter
Zjn_sc00027.1.g00760.1.sm.mk	6.861140978	-
Zjn_sc00147.1.g00830.1.am.mk	6.506860545	PF00249:Myb-like DNA-binding domain

Zjn_sc00061.1.g01390.1.am.mk	6.423035385	-
Zjn_sc00001.1.g11530.1.am.mk	6.395054587	Transferase family
Zjn_sc00087.1.g01140.1.am.mk	6.373924331	Chalcone and stilbene synthases, N-terminal domain synthases
Zjn_sc00001.1.g08520.1.am.mk	6.276480709	-
Zjn_sc00007.1.g10580.1.am.mk	6.213503135	-
Zjn_sc00029.1.g01060.1.am.mk	6.084428	--
S2 vs S1		
Zjn_sc00330.1.g00010.1.sm.mkhc	7.090687593	ABC1 family
Zjn_sc00060.1.g00600.1.am.mk	6.957861141	-
Zjn_sc00012.1.g03460.1.am.mk	6.892605828	-
Zjn_sc00015.1.g03490.1.am.mkhc	6.748853752	Alpha-amylase C-terminal beta-sheet domain
Zjn_sc00044.1.g01520.1.am.mk	6.583333202	PHD-zinc-finger like domain
Zjn_sc00015.1.g01643.1.br	6.434631372	-
Zjn_sc00037.1.g02950.1.am.mk	6.304302437	-
Zjn_sc00008.1.g07420.1.am.mk	5.802741906	-
Zjn_sc00015.1.g01646.1.br	5.755534464	Retrotransposon gag protein
Zjn_sc00043.1.g02190.1.sm.mkhc	5.71591855	-
